# Chronic Myeloid Leukemia Unveils Its Dark Side: A Rare Case of Megakaryocytic Blast Crisis

**DOI:** 10.7759/cureus.67412

**Published:** 2024-08-21

**Authors:** Mehreen Khalid, Maymoona Suhail, Alizah Faisal, FNU Poombal, Fatima Muhammad Asad Khan

**Affiliations:** 1 Hematopathology, Armed Forces Institute of Pathology, Rawalpindi, PAK; 2 Hematology, Armed Forces Institute of Pathology, Rawalpindi, PAK; 3 Hematology, Rawalpindi Medical University, Rawalpindi, PAK; 4 Pathology, Nishtar Medical University, Multan, PAK; 5 Medicine, Dow University of Health Sciences, Karachi, PAK

**Keywords:** translocation 9 22, tyrosine kinase inhibitors, acute myeloid leukemia (aml), megakaryoblastic crisis, chronic myeloid leukemia (cml)

## Abstract

Chronic myeloid leukemia (CML) can progress from a chronic phase (CP) to an accelerated phase (AP) or an acute leukemia-like blastic phase (BP). However, transformation into a megakaryoblastic phase is very rare, and such a progression is clinically significant due to its poor prognosis and resistance to standard tyrosine kinase inhibitors (TKIs). This report discusses a case of CML that progressed to a megakaryoblastic phase and the patient’s death within a month despite receiving one cycle of daunorubicin, cytarabine, and TKI chemotherapy.

A 39-year-old female with CML (CP) initially achieved hematological remission with nilotinib but later presented with B symptoms and cytopenias indicative of disease progression. A complete diagnostic workup was performed, including blood counts, bone marrow examination, flow cytometry, fluorescence in-situ hybridization (FISH), and cytogenetic testing. Peripheral blood and bone marrow evaluation confirmed blast crisis with 84% medium to large-sized blasts with basophilic cytoplasm and cytoplasmic blebs. The blasts were positive for CD41 and CD61 by immunohistochemistry (IHC). The blasts also expressed CD45 (dim), CD34, CD33, CD117, CD41, and CD61 by flow cytometry. While BCR-ABL1 positivity is typically associated with CML (90-95%), the additional findings point towards a transformation to acute megakaryoblastic leukemia (AMKL or AML-M7). The rare instance of CML's transformation to AMKL highlights the need for megakaryocytic markers in diagnostic panels to ensure accurate diagnosis and timely, tailored therapies for improved outcomes.

## Introduction

Chronic myeloid leukemia (CML) is a slow-growing blood and bone marrow disorder that typically affects individuals over their middle age, with rare cases reported in children [1]. In most CML cases, normal bone marrow cells are replaced by abnormal cells with the Philadelphia chromosome, which is characterized by the 9;22 translocations and occurs in 90-95% of patients [2]. The BCR-ABL oncogene creates a protein (tyrosine kinase) that fuels CML cell growth [3]. CML can progress through a biphasic or triphasic pattern, starting from an initial chronic phase (CP), advancing through an intermediate accelerated phase (AP), and culminating in an acute leukemia-like blastic phase (BP) [4]. Megakaryocytic blast crisis (MKBC) is marked by >20% bone marrow or peripheral blood blasts expressing megakaryocytic markers (e.g., CD41, CD61) associated with platelet function of adhesion and aggregation. MKBC is a rare entity, accounting for <3% of CML in transformation [5].

CML in blast crisis confers a dismal prognosis with a shorter survival rate compared to earlier phases [6]. This phase presents a significant therapeutic hurdle due to its aggressiveness and refractoriness to standard tyrosine kinase inhibitors (TKIs). Its management often requires a multi-pronged approach, potentially combining chemotherapy, TKIs, and allogeneic stem cell transplantation, tailored to individual response and health status [7]. We present a rare case involving the transformation of CML to a megakaryocytic blast phase. We discuss the patient's clinical course, diagnostic workup, and treatment response.

## Case presentation

History of presentation

A 39-year-old female with a documented BCR-ABL1-positive CML diagnosed in February 2021 presented with a one-month history of progressive fatigue and loss of appetite. She received nilotinib 600mg/day initially, achieving hematologic remission. However, nine months later, she experienced cytopenias and B symptoms (constitutional symptoms) prompting readmission.

Physical examination

Physical examination revealed pallor with unremarkable systemic findings.

Laboratory findings

**Figure 1:**
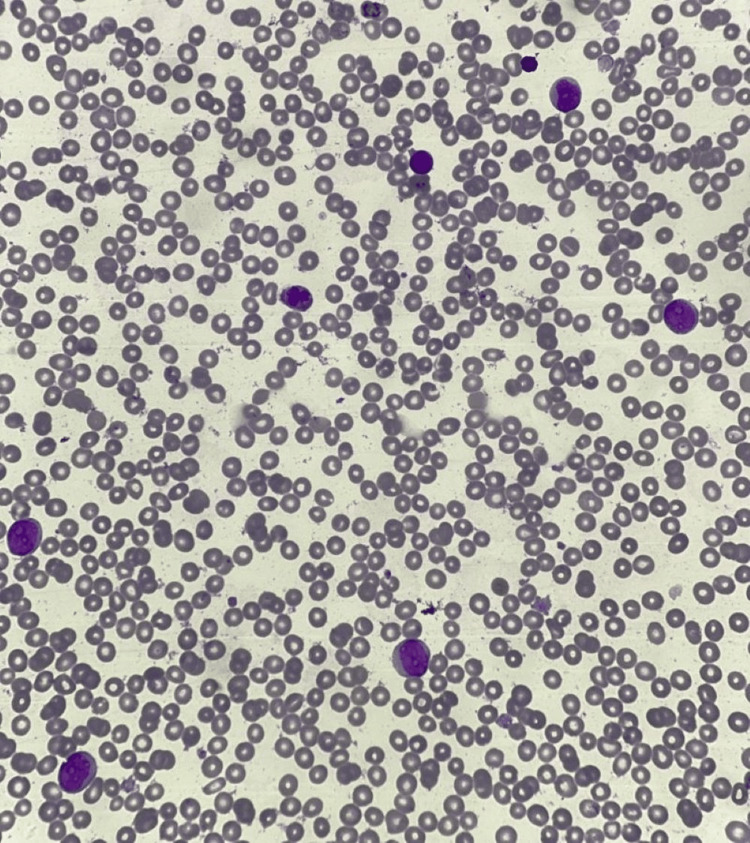
Peripheral smear at 40X showing blasts

Peripheral blood smear analysis revealed normocytic normochromic anemia with many teardrop-shaped red blood cells. Laboratory findings are presented in Table 1. Morphologically, the blasts exhibited round nuclei with open chromatin, prominent nucleoli, and deep basophilic cytoplasm whereas the platelets were giant and showed anisocytosis, as shown in Figure 1.

**Table 1:** Laboratory findings

Lab tests	Patients values	Reference values
Complete blood count		
Haemoglobin (g/dl)	11.4	12-15
Platelet count (x10^9^/L)	50	150-450
Total leukocyte count (x10^9^/L)	25.19	4-10
Differential count		
Neutrophils (%)	0	40-80
Lymphocytes (%)	20	20-40
Monocytes (%)	2	2-10
Eosinophils (%)	0	1-6
Myelocytes (%)	3	0
Blast cells (%)	75	0

Bone marrow evaluation

**Figure 2:**
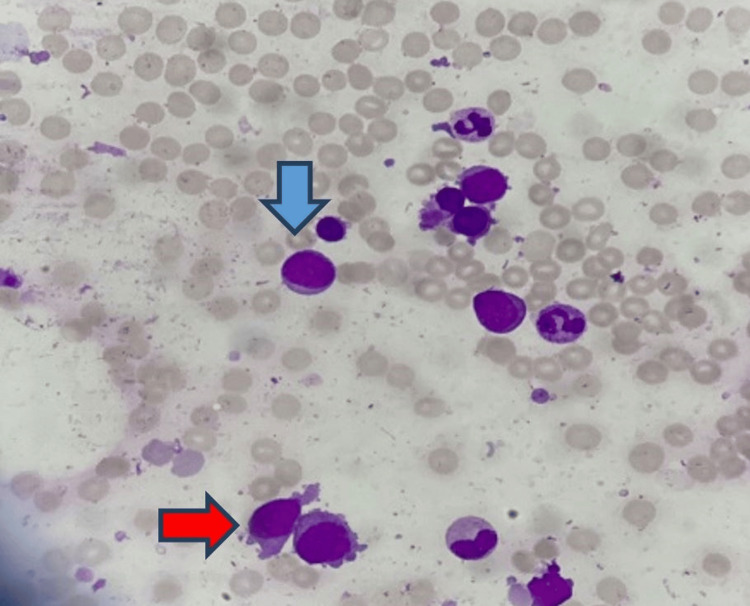
Bone marrow aspirate at 40X showing blasts with blebs/projections (megakaryoblasts) and occasional myeloblasts (blue arrow: myeloblast, red arrow: megakaryoblast)

**Figure 3:**
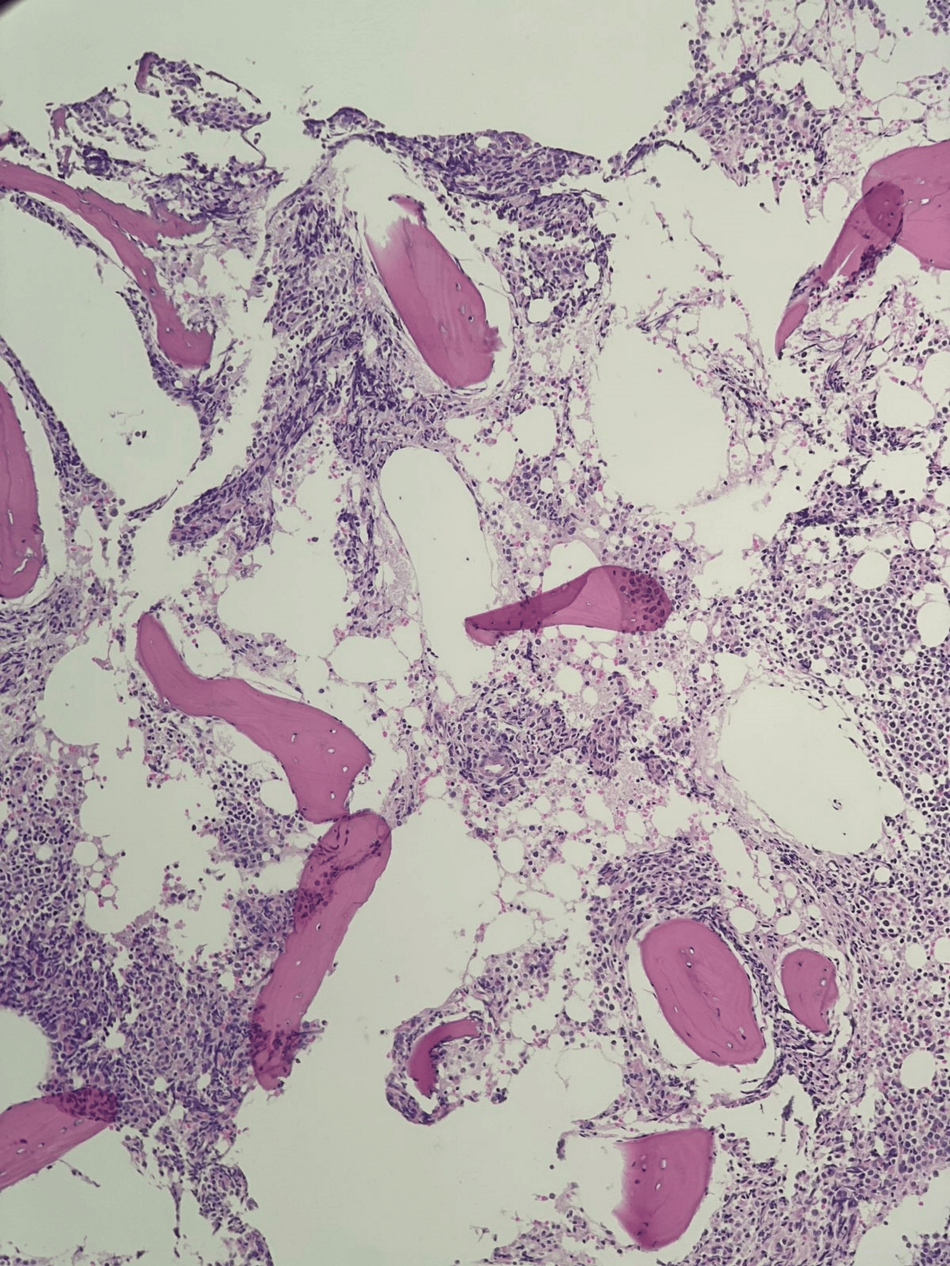
H&E-stained trephine biopsy at 10X showing hypercellular marrow with effaced architecture and diffuse infiltration by blast cells

**Figure 4:**
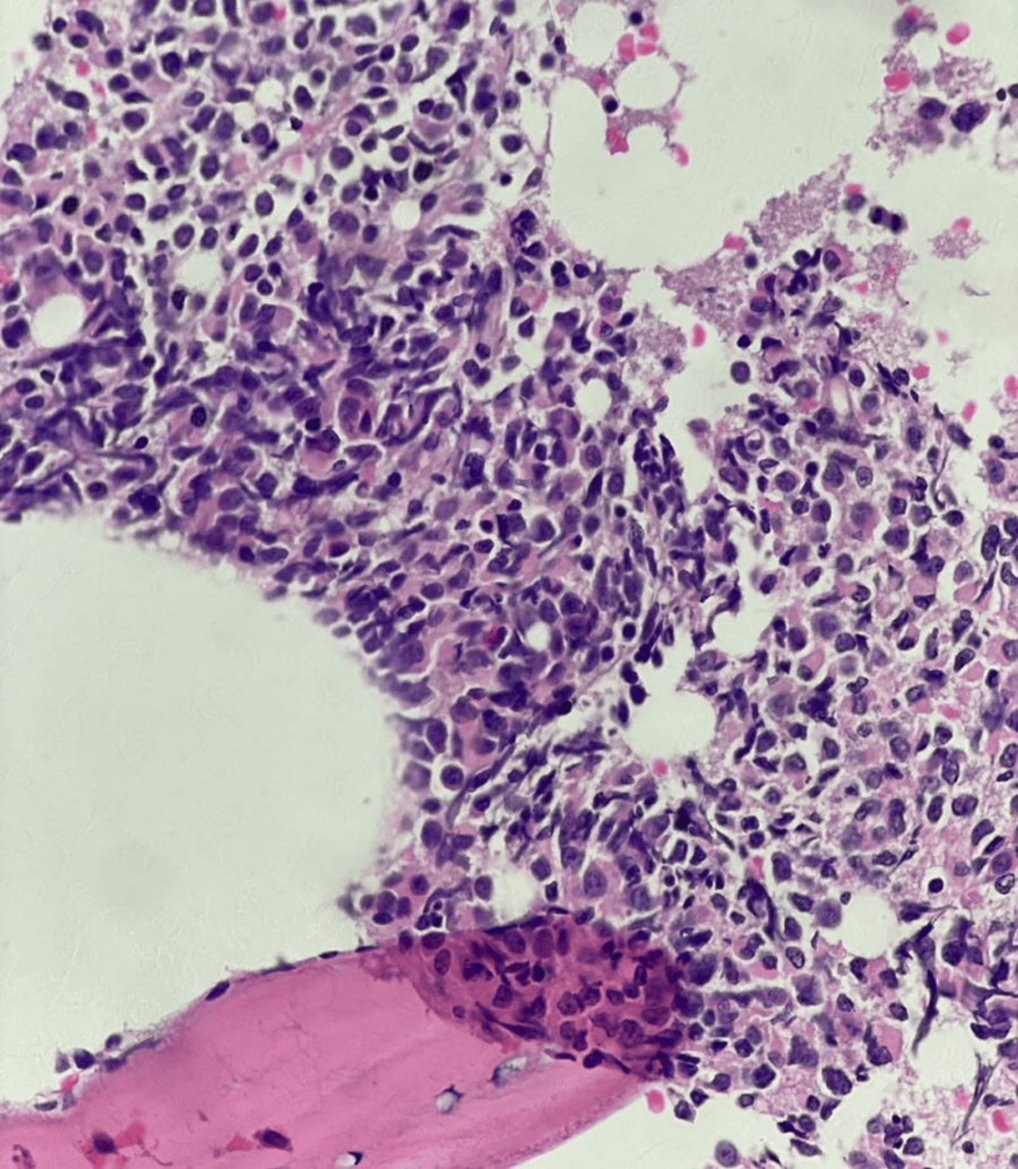
H&E-stained trephine biopsy at 40X showing diffuse infiltration by blast cells

**Figure 5:**
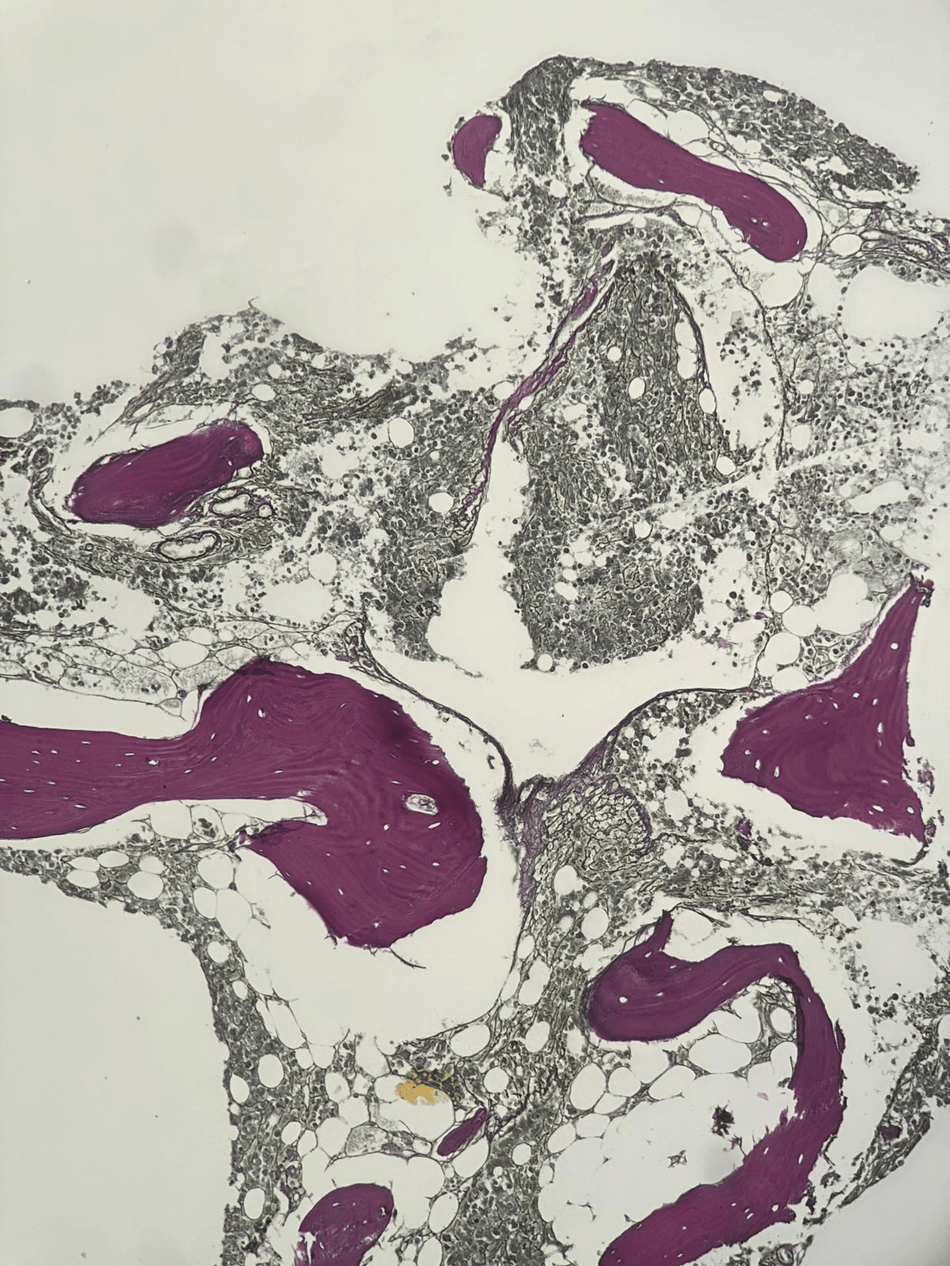
Increased reticulin at 10X indicating extensive fibrosis (MF grade 2)

Aspiration from the posterior iliac crest revealed hypercellular fragments and trails with depressed erythropoiesis and myelopoiesis. Megakaryocytes were increased in number and there were 84% blast cells. The population of the blast cells was heterogeneous, consisting of medium to large-sized blast cells with a low nuclear-to-cytoplasmic ratio, dispersed chromatin, conspicuous nucleoli, and abundant cytoplasm. The majority of the blast cells had cytoplasmic blebbing (Figure 2). Trephine biopsy showed infiltration with sheets of blast cells (Figures 3-4) and extensive fibrosis (Figure 5).

**Figure 6:**
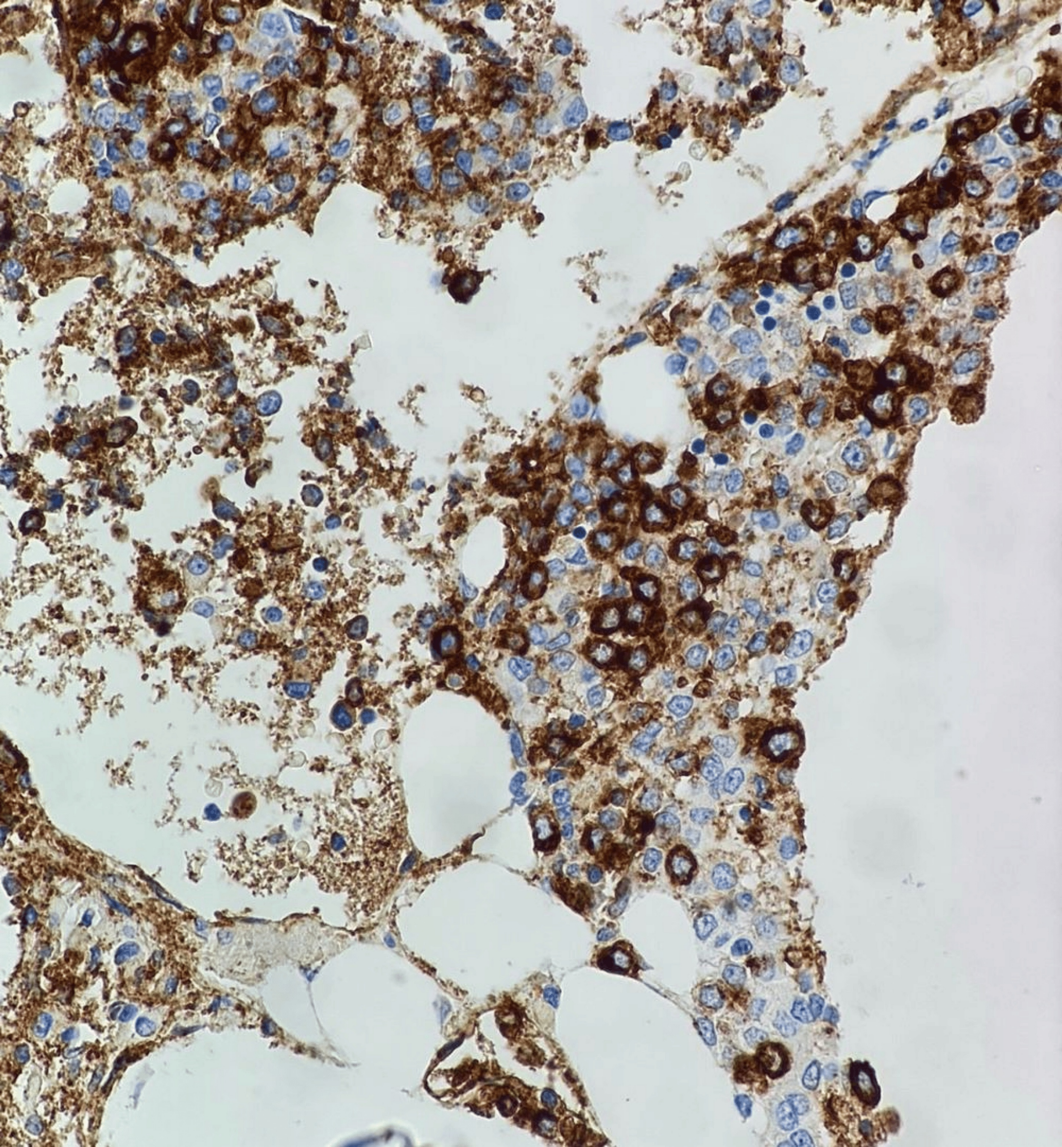
Immunohistochemistry (IHC): CD41 at 40X showing positive staining of megakaryoblasts

**Figure 7:**
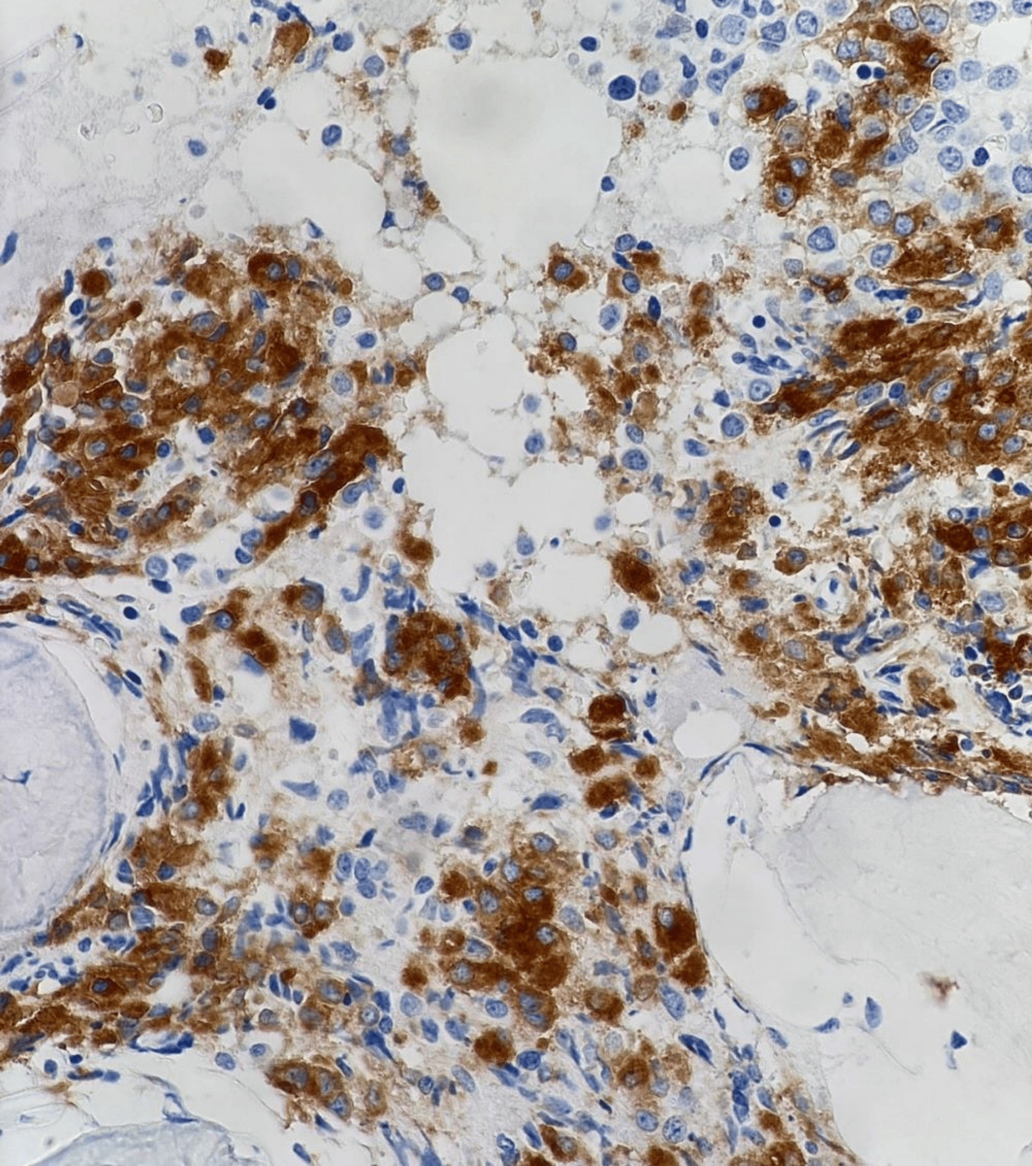
Immunohistochemistry (IHC): CD61 at 40X showing positive staining of megakaryoblasts

Flow cytometry and immunohistochemistry confirmed a population of blasts expressing CD45 (dim), CD34, CD33, CD117, CD41 (Figure 6), and CD61 (Figure 7). Additionally, HLA-DR was also expressed. Quantitative PCR (polymerase chain reaction) for BCR-ABL1 confirmed a positive result (41.39% IS). Cytogenetic analysis revealed the presence of the characteristic t(9;22) translocation and a complex karyotype.

Diagnosis

Based on the clinical presentation, laboratory findings, bone marrow evaluation, and molecular analysis, the diagnosis of chronic myeloid leukemia in megakaryocytic blast crisis was established.

Treatment

Unfortunately, the patient passed away four months after receiving one cycle of chemotherapy. While we do not currently have details on the specific type of chemotherapy administered, we have requested the patient's family for this information.

## Discussion

The abnormal fusion of two genes, BCR (on chromosome 22) and ABL1 (on chromosome 9) through reciprocal translocation, t(9;22)(q34;q11), results in the BCR-ABL1 fusion protein [8]. The presence of the BCR-ABL1 fusion gene in hematopoietic stem cells is adequate to trigger CML. The shortened chromosome 22, termed the Philadelphia chromosome, contains the BCR-ABL1 oncogene, a significant player in the leukemogenesis of CML due to its deregulated tyrosine kinase activity [9]. BCR-ABL1 is also present in other leukemia types, including acute lymphoblastic leukemia (ALL) and acute myeloid leukemia (AML) [10].

As discussed previously, CML is classified into three phases: CP, AP, and BP [11]. Annually, about 1-1.5% of CML patients treated with a TKI progress to an advanced phase or blast crisis [12]. Two-thirds of CML blast phase cases exhibit a myeloid phenotype; the remaining cases present a lymphoid phenotype, though blast transformation into a megakaryocytic phenotype is rare. Immunophenotypic analysis helps classify the blast nature. Extramedullary blast crisis may affect various sites and manifest as either lymphoid or myeloid [13].

The blast phase of CML is a poor prognostic factor. Other prognostic factors include age (60 years and older), platelet count, increased basophil counts, blasts in the blood, and spleen size. These factors are integrated into prognostic scoring systems such as Sokal, European Treatment and Outcome Study for CML (EUTOS) long-term survival (ELTS), and Hasford, which determine the risk profile of CP-CML patients at diagnosis [14]. Despite significant advancements in managing the CP phase of CML, the blast crisis continues to present significant therapeutic challenges, underlining the need for novel therapies to induce a second CP, thereby facilitating the transition to allogeneic stem cell transplantation [15].

CML megakaryocytic blast crisis typically has a poor prognosis [16]. Detecting the specific type is crucial as management protocols differ. In our case, the patient's bone marrow aspiration showed diluted marrow, likely associated with myelofibrosis, a common occurrence in both de novo acute megakaryocytic leukemia (AMKL or AML-M7) and megakaryocytic blast crisis [17]. Diagnosing unusual blast forms presents challenges but is essential for appropriate management. Patients with megakaryocytic blast phase CML have a poor prognosis, with a median survival rate of less than 12 months [16].

## Conclusions

AML-M7 blastic crisis in CML is a very rare manifestation, and the detection of this condition is crucial as the management protocols differ. The case report highlights the significance of distinguishing the CML blast crisis from other similar differentials. This case also emphasizes the importance of extensive diagnostic evaluations to accurately identify rare subtypes of blast crisis. Including one megakaryocytic marker in the initial flow cytometry panel is crucial to prevent underdiagnosis as AML alone and ensure appropriate treatment decisions. Recognizing the rare AML-M7 type in a blast crisis can lead to more effective therapeutic involvement and improve patient outcomes.

## References

[1] Chronic Myelogenous Leukemia Treatment (2024). Chronic myelogenous leukemia treatment. https://www.cancer.gov/types/leukemia/patient/cml-treatment-pdq.

[2] Wang Z, Zen W, Meng F (2015). Chronic myeloid leukemia with variation of translocation at (Ph) [ins (22;9) (q11;q21q34)]: a case report. Int J Clin Exp Pathol.

[3] Amarante-Mendes GP, Rana A, Datoguia TS, Hamerschlak N, Brumatti G (2022). BCR-ABL1 tyrosine kinase complex signaling transduction: challenges to overcome resistance in chronic myeloid leukemia. Pharmaceutics.

[4] Giles FJ, Cortes JE, Kantarjian HM, O'Brien SM (2004). Accelerated and blastic phases of chronic myelogenous leukemia. Hematol Oncol Clin North Am.

[5] Agrawal S, Kumar K, Singh M, Chandra H (2022). Megakaryocytic blast crisis in chronic myeloid leukiemia: an uncommon presentation in a common neoplasm. Hematol Transfus Cell Ther.

[6] Karkuzhali P, Shanthi V, Usha T (2013). A case of chronic myeloid leukaemia presenting as megakaryocytic blast crisis (AML M7). Ecancermedicalscience.

[7] Thompson PA, Kantarjian HM, Cortes JE (2015). Diagnosis and treatment of chronic myeloid leukemia in 2015. Mayo Clin Proc.

[8] Khemka R, Gupta M, Jena NK (2019). CML with megakaryocytic blast crisis: report of 3 cases. Pathol Oncol Res.

[9] Quintás-Cardama A, Cortes J (2009). Molecular biology of bcr-abl1-positive chronic myeloid leukemia. Blood.

[10] Kang ZJ, Liu YF, Xu LZ (2016). The Philadelphia chromosome in leukemogenesis. Chin J Cancer.

[11] Jabbour E, Kantarjian H (2020). Chronic myeloid leukemia: 2020 update on diagnosis, therapy and monitoring. Am J Hematol.

[12] Kirwin M, Yee J (2020). Blast crisis. J Educ Teach Emerg Med.

[13] (2018). Chronic myeloid leukemia. Hematology (Seventh Edition).

[14] Specchia G, Pregno P, Breccia M (2021). Prognostic factors for overall survival in chronic myeloid leukemia patients: a multicentric cohort study by the Italian CML GIMEMA Network. Front Oncol.

[15] Yohanan B, George B (2022). Current management of chronic myeloid leukemia myeloid blast phase. Clin Med Insights Oncol.

[16] Jain P, Kantarjian HM, Ghorab A (2017). Prognostic factors and survival outcomes in patients with chronic myeloid leukemia in blast phase in the tyrosine kinase inhibitor era: cohort study of 477 patients. Cancer.

[17] Zhao G, Wu W, Wang X, Gu J (2018). Clinical diagnosis of adult patients with acute megakaryocytic leukemia. Oncol Lett.

